# *Clostridium butyricum* Probiotic Feed Additive: Modulation of Sow Milk Metabolomics and Mitigation of Pre-Weaning Piglet Diarrhea

**DOI:** 10.3390/ani14142098

**Published:** 2024-07-18

**Authors:** Jakavat Ruampatana, Junpen Suwimonteerabutr, Kunaporn Homyog, Wanwimon Mekboonsonglarp, Korntip Kanjanavaikoon, Wouter Van der Veken, Sutthasinee Poonyachoti, Takele Feyera, Sarn Settachaimongkon, Morakot Nuntapaitoon

**Affiliations:** 1 Department of Obstetrics, Gynaecology and Reproduction, Faculty of Veterinary Science, Chulalongkorn University, Bangkok 10330, Thailand; jakavat.r@gmail.com (J.R.);; 2 Center of Excellence in Swine Reproduction, Department of Obstetrics, Gynaecology and Reproduction, Faculty of Veterinary Science, Chulalongkorn University, Bangkok 10330, Thailand; 3 Center of Veterinary Diagnosis, Faculty of Veterinary Science, Mahidol University, Nakhon Pathom 73170, Thailand; 4 Scientific and Technological Research Equipment Center (STREC), Chulalongkorn University, Bangkok 10330, Thailand; 5 Huvepharma (Thailand) Co., Ltd., Bangkok 10900, Thailand; 6 Huvepharma N.V., 2600 Antwerp, Belgium; 7 Department of Physiology, Faculty of Veterinary Science, Chulalongkorn University, Bangkok 10330, Thailand; 8 Department of Animal Science and Veterinary Sciences, Aarhus University, AU-Viborg, DK-8830 Tjele, Denmark; 9 Department of Food Technology, Faculty of Science, Chulalongkorn University, Bangkok 10330, Thailand; 10 Omics Sciences and Bioinformatics Center, Faculty of Science, Chulalongkorn University, Bangkok 10330, Thailand

**Keywords:** probiotics, feed additives, metabolomics, lipidomics, sow milk, piglet performance

## Abstract

**Simple Summary:**

Pre-weaning piglet diarrhea is one of the major concerns in the swine industry. Recently, various probiotics have been applied to improve animal gut health and performance. This study investigated the impact of *C. butyricum* probiotic feed additive on sow and piglet performance, together with alterations in lipidomic and metabolomic profiles of sow milk. Results showed that sows given the probiotics had lower backfat loss and their piglets experienced less diarrhea incidence, although there were no other significant benefits for piglet growth. Changes in certain fatty acids and metabolites present in sow colostrum and milk, which could impact their nutritional profiles and the health of the piglets, were significantly observed. This study provided new insights regarding the impacts of probiotics application that could potentially lead to better outcomes in swine farms.

**Abstract:**

The present study aimed to investigate the impact of *Clostridium butyricum* probiotic feed additive on sow and piglet performances, together with alterations in the lipidomic and metabolomic profiles of sow milk. Sixty-four Landrace × Yorkshire crossbred sows and 794 piglets were included. Sows were divided into two groups; i.e., (i) conventional gestation diet (control; *n* = 35) and (ii) conventional diet added with 10 g/sow/day of probiotic *C. butyricum* spores (treatment; *n* = 29) from one week before the estimated farrowing day until weaning (29.6 ± 4.8 days). The sow and piglet performances and incidence of piglet diarrhea were recorded. Changes in gross chemical composition, fatty acid and non-volatile polar metabolite profiles of sow colostrum, transient milk and mature milk were evaluated. The results showed that relative backfat loss in the treatment group (−2.3%) was significantly lower than in control group (11.6%), especially in primiparous sows (*p* = 0.019). The application of *C. butyricum* probiotics in sows significantly reduced the incidence of diarrhea in piglets (*p* < 0.001) but no other effect on piglet performance was found. Lipidomic and metabolomic analyses revealed variations in sow colostrum and milk biomolecular profiles, with indicative compounds significantly altered by feeding with the *C. butyricum* probiotics. In conclusion, the use of *C. butyricum* probiotics in sows may improve sow body condition and reduce diarrhea incidence in piglets, with underlying changes in milk composition that warrant further investigation. These findings support the potential of *C. butyricum* as a beneficial feed additive in swine production.

## 1. Introduction

Pre-weaning piglet diarrhea is well recognized as a major concern in the swine industry. The diarrhea causes malabsorption and excessive secretion of water and electrolytes into the intestine resulting in watery feces, nausea, abdominal cramps, shivering, decreased feed intake, growth retardation and increased piglet mortality [1]. Several pathogens—as causative agents for pre-weaning piglet diarrhea—include *Escherichia coli*, *Enterococcus hirae, Clostridium difficile*, *Clostridium perfringens*, *Salmonella* spp., *Campylobacter* spp., rotavirus, coronavirus and *Cryptosporidium* spp. [2]. In addition, the use of antibiotics, various preventive measures and applications of feed additives have been alternatively introduced to improve intestinal health and reduce the incidence of diarrhea in newborn piglets.

Probiotics are defined as live microorganisms, which confer a health benefit on the host, when administered in adequate amounts [3]. Probiotics have been introduced as alternative feed additives, aiming to reduce antimicrobial resistance and drug residues in the swine production chain [4]. *Clostridium butyricum* (*C. butyricum*)—a Gram-positive obligate anaerobic bacillus—has been acknowledged for its probiotic capacity; i.e., modulation of gut microbiota, improvement of intestinal barrier functions or protection against pathogenic bacteria, which enhance growth and reduce diarrhea incidence in piglets [5]. The principal effects of *C. butyricum* probiotics are associated with its ability to produce short-chain fatty acids (especially butyric acid), amino acids, enzymes, and vitamins which play a crucial role in energy metabolism and the development of healthy intestinal epithelial cells [5]. In addition, *C. butyricum* feed additives are reported to be associated with enhanced digestibility and nutrient absorption of pigs [4]. Therefore, many studies focused on the influence of the presence of *C. butyricum* probiotics in the feed of a sow herd—especially during late-gestation and lactation period—on the production and quality of colostrum and milk of the sows [6,7,8]. It is well recognized that sow colostrum and milk are essential sources of energy, passive immunity and nutrients that support the growth and survival of newborn piglets during the lactation period [9]. Various strategic dietary supplements aiming to ameliorate yields, biochemical and immunological composition in sow colostrum and milk have been attempted [10,11,12]. Regarding the effects of dietary probiotics, alterations in gross chemical composition—i.e., fat, protein, lactose, milk-solids-not-fat, IgA and IgG—in sow colostrum and milk have been indicated in many studies [6,7,13,14]. Nevertheless, changes in minor milk components affected by dietary probiotic intake have not been well investigated.

Metabolomics—a comprehensive characterization of small molecular weight metabolites (<1.5 kDa) present in biological matrices—has recently been acknowledged in lactation biology, milk and dairy research [15]. This high-throughput approach allows a better understanding of dynamic changes in milk metabolome, influenced by various inherent and environmental factors in dairy production [15]. Although metabolomics has been applied in swine milk research [16,17,18], publications using this approach to investigate the impact of probiotic feed additives on the alteration in sow milk metabolome are rather limited [19]. This information could provide a better understanding and novel insights into the relationships between probiotic-induced changes in sow milk composition and piglet performance. 

Therefore, the aims of this study were to investigate the impact of dietary *C. butyricum* probiotic administration during the late-gestation and lactation period on: (i) changes in the performances of lactating sows and pre-weaning piglets; along with (ii) the alterations in fatty acid and non-volatile polar metabolite profiles of sow colostrum and milk.

## 2. Materials and Methods

### 2.1. Animal Care

All experimental protocols in this study were approved by the Institutional Animal Care and Use Committee (IACUC) at the Faculty of Veterinary Science, Chulalongkorn University (Approval number 2031056) and followed the guidelines documented in “The ethical Principles and Guidelines for the Use of Animals for Scientific Purposes” edited by the National Research Council of Thailand.

### 2.2. Experimental Design

The study was carried out in a commercial swine farm in the western part of Thailand. Sixty-four crossbred Landrace × Yorkshire F1 sows, with parities between 1 and 7; and 794 piglets were included in this study (control group; *n* = 426 and treatment group; *n* = 368). All sows were raised in a conventional open-housing system in the same week. Sows were transferred to the farrowing house seven days before the estimated farrowing date and were housed individually in crates until weaning. Additionally, backfat thickness was measured when sows were introduced to the farrowing house at day 109 of gestation and weaning, using A-mode ultrasonography (Renco Lean-Maeter^®^, Minneapolis, MN, USA). Animals were divided into two groups according to feeding regimen; i.e., (i) conventional gestation diet (control; *n* = 35) and (ii) conventional diet incorporated with 10 g/sow/day (5 × 10^9^ CFU) of probiotic *C. butyricum* spores (Top Gut^®^, Huvepharma Ltd., Bangkok, Thailand) (treatment; *n* = 29) by top dressing from a week before the estimated farrowing day until weaning (29.6 ± 4.8 days). The *C. butyricum* was authorized as a zootechnical feed additive for pigs (Regulation (EU) No. 2021/1411) [20]. Sow performance parameters recorded during the experiment included: parity number; number of total born piglets per litter; live-born piglets per litter; percentage of stillborn piglets; percentage of mummified fetuses; number of weaned piglets per litter; litter weight at 3, 10, 17, 21 days and weaning; backfat thickness at day 109 of gestation, and weaning; and backfat loss. Piglets were identified by number. The incidence of diarrhea in piglets was determined by observation of their fecal characteristics throughout the entire lactation period. The diarrhea score was assigned as normal feces (score = 0), soft (score = 1) and runny and/or watery feces (score = 2). Piglet body weight was measured at birth and subsequently on days 1, 3 and 21 of lactation. 

### 2.3. Animals, Housing and Management

The experiment was conducted in a commercial farm in Thailand. The number of productive sows was 3000. Sows were kept in a conventional evaporative cooling system. Sows were housed in individual pens (1.50 m^2^) during gestation and were fed a commercial gestation diet according to requirements. Feed was provided twice a day following a standardized feeding pattern, resulting in an average of 3 kg of feed per sow, daily. The water was supplied ad libitum from individual nipple drinkers. On day 109 of gestation, sows were moved to the farrowing house. 

The farrowing facilities were an evaporation housing system. Each sow was housed in an individual farrowing pen (2.95 m^2^) distributed in 3 rows, with a central alley for sows and 2 side alleys for piglets. The farrowing pens were slatted, with concrete at their center for the sows; and were equipped with steel slats on both sides, for the piglet. Each farrowing pen included a creep area for the piglets (0.60 m^2^) on one side, covered by a plastic plate and equipped with a heating lamp, a rubber mattress, and a feeding bowl. Lactating sows were fed twice a day with a dry corn-soybean meal diet that met or exceeded nutritional requirements [21]. The nutritional content of the experimental diet in the present study is presented in Table 1. The amount of feed offered was increased daily until libitum feed was reached after one week of lactation. Sows and piglets had ad libitum access to water via separated nipple drinkers. 

The farrowing process was carefully supervised. During farrowing, the sows and piglets were interfered with as little as possible. Routine interventions were limited to visual supervision of farrowing and the removal of placenta, mummified piglets or dead piglets. Farrowing assistance was provided by skilled personnel when the birth interval exceeded 45 min, and/or there was no progress of uterine contraction. Newborn piglets were dried with a towel before being numbered. No extra management was performed on the newborn piglets. Routine procedures for piglets involved tail docking, tooth clipping, and administering a 1 mL iron supplement intramuscularly (ABI-DEX^®^ 100, T.P. Drug laboratories (1996) Co., Ltd., Bangkok, Thailand) on their first day of life. On the third day, piglets received an oral dose of coccidiocide (Baycox^®^, OLIC Co., Ltd., Ayutthaya, Thailand). Throughout the entire study, the animals were checked daily for health or eating problems. No pathological symptoms were observed on the farm during the study.

### 2.4. Determination of Colostrum Consumption and Colostrum Yield

Colostrum consumption of individual piglets was estimated using the equation previously proposed by Theil et al. [22]. The colostrum yield was defined as the sum of individual colostrum consumption by all piglets in the litter. Milk yield was calculated using the Bayesian hierarchical model previously reported by Hansen et al. [23].

### 2.5. Collection of Colostrum and Milk Samples

Colostrum was manually collected by hand from all functional teats within one hour after the onset of farrowing. Transient and mature milk was collected from all functional teats on days 3 and 10 of lactation, respectively. For the transient and mature milk collections, sows received an intravenous injection of 0.2 mL oxytocin (10 IU/mL, VetOne^®^, Boise, ID, USA) to facilitate milk let-down. Before colostrum and milk collection, all udders were cleaned with sterile water and dried with a towel to reduce contamination. Approximately 30 mL of colostrum, transient and mature milk were collected from all functional mammary glands of the sows, into plastic cups. The samples were filtered through gauze, transferred into a clean bottle (30 mL), and stored in a cool Styrofoam box (4 °C) during the collection. Once the samples arrived at the laboratory, milk samples were centrifuged at 2700× *g* for 20 min at 4 °C (Centrifuge 5810R, Eppendorf SE, Hamburg, Germany) and stored at −20 °C until further analysis. 

### 2.6. Determination of Major Chemical Composition in Sow Colostrum and Milk 

The major chemical composition—i.e., fat, protein, lactose, dry matter and casein concentration (%wt/wt)—of colostrum and milk samples were analyzed using infrared spectroscopy (MilkoScan FT2 instrument, Foss MilkoScan, Hillerød, Denmark). The concentrations of IgG and IgA in colostrum samples were determined according to the methods described in our previous work [24].

### 2.7. Analysis of Fatty Acid Profiles in Sow Colostrum and Milk 

Fatty acid (FA) composition in sow colostrum and milk samples were characterized using gas chromatography coupled with mass spectrometry for fatty acid methyl ester (GC-MS-FAME) analysis (Agilent 7890A-5975C, Agilent Technologies, Santa Clara, CA, USA) according to the method used in our previous study [18]. In brief, fatty acid methyl ester (FAME) formation was initialized after heating and hydrolysis of samples with KOH, MeOH and H_2_SO_4_. The FAME fractions were collected after hexane extraction. FA composition of the FAME fraction was determined by capillary GC on a SP-2560 capillary column (Supelco, Bellefonte, PA, USA) operated using similar parameters as described previously [18]. FAs were identified by comparing their specific retention time and *m/z* model with a fatty acid methyl ester standard (Supelco 37 Component FAME mix, Sigma-Aldrich, Steinheim, Germany). After automated peak integration, the concentrations of FAs were calculated using calibration curves fitted by a linear regression model and finally expressed as mg/100 g [18]. 

### 2.8. Analysis of Non-Volatile Polar Metabolite Profiles in Sow Colostrum and Milk

Non-volatile polar metabolite composition in sow colostrum and milk samples were characterized using non-targeted proton nuclear magnetic resonance (^1^H-NMR) metabolomics, according to the method used in our previous work [18]. In brief, the pH of samples was adjusted to 6.0. Lipid and large protein fractions were removed by dichloromethane extraction and ultra-centrifugation (74200× *g* for 60 min at 4 °C), respectively. The supernatant serum was then ultra-filtrated through a Pall Nanosep^®^ centrifugal device with 3 kDa molecular weight cutoffs (Pall Life Sciences, Ann Arbor, MI, USA). Finally, the clear milk serum was mixed 1:1 (*v*/*v*) with a phosphate buffer pH 6.0 consisting of 1 mM 3-(Trimethylsilyl) propionic-2, 2, 3, 3-d4 acid sodium salt (TSP) (Merck, Darmstadt, Germany) as an internal standard. Samples were then subjected to a 500 MHz NOESY-GPPR-1D-^1^H-NMR spectrometer (Bruker, Rheinstetten, Germany) operated with similar parameters as described in our previous study [18]. ^1^H-NMR spectra were corrected, pre-treated, and segmented using a binning technique. Metabolite identification was performed by consulting the Chenomx NMR suite 8.2 library (Chenomx Inc., Edmonton, AB, Canada), Livestock Metabolome Database (www.lmdb.ca; accessed on 12 August 2023), and literature sources [16,18,25,26]. The sum of signal intensity corresponding to respective metabolites was expressed in arbitrary units. The ^1^H-NMR signal intensities of respective compounds were expressed as log_10_ transformed [arbitrary unit] and introduced as variables in the statistical analysis [18]. 

### 2.9. Statistical Analysis

Descriptive statistics (i.e., mean, standard deviation and range) were performed using SAS version 9.4 (SAS Institute Inc., Cary, NC, USA). Sow performances were analyzed via multiple analyses of variance, using the general linear model procedure of SAS. Piglet performance (i.e., body weight at 0, 1, 3 and 21 days of age) were analyzed by using the general linear mixed model procedure of SAS. For all analyses, the statistical models included the fixed effect of the group (control and treatment group), parity classes (1, 2–4 and 5–7) and the interaction between group and parity classes. Sow identity was included in the models as a random effect. The effect of the probiotic feed additive on the incidence of piglets’ diarrhea on each day was analyzed by the Wilcoxon Rank Sum test. A general linear model procedure was used to analyze the effects of the group (control and treatment group), parity classes (1, 2–4 and 5–7) and the interaction between group and parity classes on colostrum and milk composition on days 3 and 17 (on each day). Least-squares means were obtained from each class of the parity. The probability at *p* < 0.05 was regarded to be statistically significant.

GC-derived lipidomic and ^1^H-NMR-derived metabolomic data were pretreated, normalized and subjected to multivariate statistical analysis in MetaboAnalyst 5.0 software (www.metaboanalyst.ca; accessed on 7 October 2023). Partial least-squares discriminant analysis (PLS-DA) was applied to visualize distinctive fatty acid and non-volatile polar metabolite patterns between control and treatment samples with important statistical parameters; i.e., % prediction accuracy, *R*^2^ and *Q*^2^ values, and variable importance in projection (VIP) scores, as described in our previous work [20].

## 3. Results

### 3.1. Sow Reproductive Performances

On average, the sow parity number was 2.6 ± 1.8. The number of total piglets born/litter and the number of live-born piglets/litter were 12.5 ± 2.9 and 10.8 ± 2.9 piglets/litter, respectively. The effects of dietary *C. butyricum* probiotic additive on sow reproductive performance are presented in Table 2. The results showed that there was no significant influence of *C. butyricum* probiotics on the reproductive performance of the sows in this study (*p* > 0.05). 

When the sow parity number was considered (Table 3), a significant difference in relative backfat loss was observed between primiparous sows in the control (11.6%) and treatment groups (−2.3%, *p* = 0.019). Sows in parity 2–4 that received the probiotic additive tended to deliver a higher litter weight at weaning (*p* = 0.149), with a lower relative backfat loss (*p* = 0.167) compared to those in the control group (Table 2). However, primiparous sows fed with the *C. butyricum* probiotics tended to yield a higher milk quantity (7.8 kg/day) compared to the control group (6.4 kg/day) (*p* = 0.181).

### 3.2. Pre-Weaning Piglet Performances

**Figure 1:**
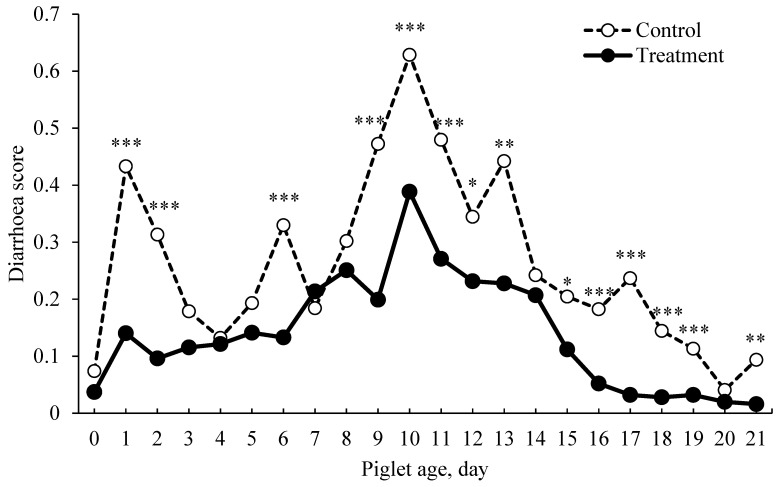
Incidence of diarrhea in piglets, from birth to 21 days in in the control and treatment groups. Level of significant difference at * *p* < 0.05, ** *p* < 0.01 and *** *p* < 0.001.

The average piglet’s birth weight and piglet’s weight at 24 h after birth were 1.48 ± 0.41 and 1.55 ± 0.41 kg, respectively. No effect of *C. butyricum* probiotic additive on pre-weaning piglet performance was found in this study (Table 4). In sow parity 5–7, the piglet weight at day 21 of lactation was higher in the treatment than in the control group (4.55 vs. 3.29 kg, *p* = 0.026) (Table 5). The incidence of diarrhea in piglets throughout the entire lactation period is shown in Figure 1. The figure revealed that piglets belonging to sows fed with the *C. butyricum* probiotics had significantly lower diarrhea scores compared to those from mothers in the control group (*p* < 0.05). 

### 3.3. Major Chemical Composition of Sow Colostrum and Milk 

There was no significant effect of *C. butyricum* probiotic additive on changes in fat, protein, lactose, dry matter and casein concentration of colostrum and milk samples obtained from sows in the treatment group of this study (*p* > 0.05) (Table 6). Similar results were found for antibody levels, IgG and IgA, in colostrum samples (Table 6).

### 3.4. Changes in Fatty Acid Profiles of Sow Colostrum and Milk

**Figure 2:**
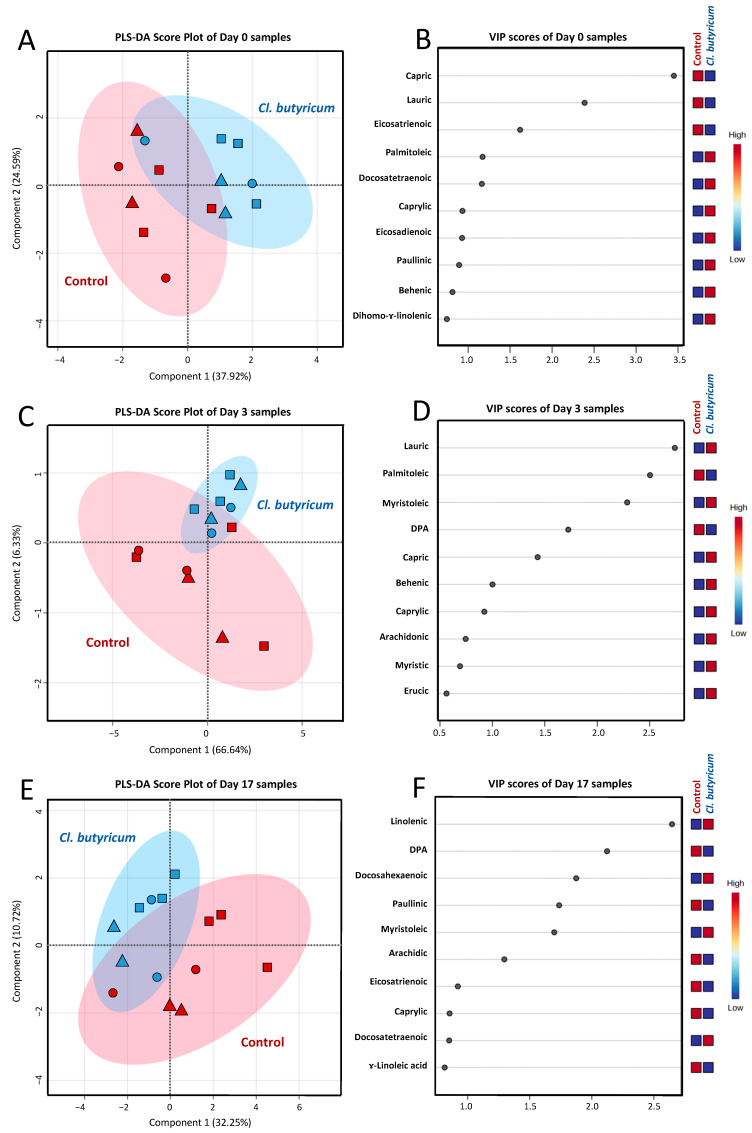
PLS-DA score plots for the comparison of fatty acid profiles of colostrum (day 0; panel (**A**)), transient milk (day 3; panel (**C**)) and mature milk (day 17; panel (**E**)) samples collected from sows in the control (red color) and *C. butyricum* treatment (blue color) groups. Samples from animals in parity 1 (■), parity 2–4 (▲) and parity 5–7 (●) are differently symbolized. VIP scores derived from the comparison among samples of the same day postpartum and indicative fatty acids accountable for the discrimination are visualized in panel (**B**), (**D**) and (**F**), respectively.

Data from our previous work have demonstrated that the day after farrowing provided a significant impact on the biomolecular profile; i.e., fatty acids and non-volatile polar metabolites, of sow colostrum and milk [20]. Therefore, the influence of dietary *C. butyricum* probiotic additive on the FA profiles of milk samples were evaluated for colostrum (day 0), transient (day 3) and mature milk (day 17), independently. Three separate PLS-DAs were performed for the comparison of samples collected within the same day (Figure 2). Regarding colostrum, PLS-DA demonstrated a good distinction pattern between the control and *C. butyricum* treatment group, with a prediction accuracy of 69.71%, *R*^2^ = 0.623 and *Q*^2^ = 0.511 (Figure 2A). VIP scores with a value greater than 1.0 were used to screen out the discriminant FAs. Results indicated that variations in the concentration of capric (C10:0), lauric (C12:0), eicosatrienoic (C20:3n3), palmitoleic (C16:1), docosatetraenoic (C22:4), caprylic (C8:0), eicosadienoic (C20:2n6) and paullinic (C20:1n7) acid were responsible for the discrimination (Figure 2B). Continuing with transient milk: a good distinction pattern between the control and *C. butyricum* treatment group was also shown by PLS-DA with a prediction accuracy of 64.29%, *R*^2^ = 0.699 and *Q*^2^ = 0.372 (Figure 2C). VIP scores indicated that variations in the concentration of lauric (C12:0), palmitoleic (C16:1), myristoleic (C14:1), DPA (22:5n3), capric (C10:0), behenic (C22:0) and caprylic (C8:0) acid were responsible for the discrimination (Figure 2D). In matured milk, a distinct discriminative pattern between the control and *C. butyricum* treatment group was still observed by PLS-DA with a prediction accuracy of 57.14%, *R*^2^ = 0.652 and *Q*^2^ = 0.449 (Figure 2E). VIP scores indicated that variations in the concentration of linolenic (C18:3n3), DPA (22:5n3), docosahexaenoic (C22:6n3), paullinic (C20:1n7), myristoleic (C14:1) and arachidic (C20:0) acid were responsible for the discrimination (Figure 2F). The statistically significant levels of indicative FAs for discrimination between the control and *C. butyricum* treatment group in colostrum, transient and mature milk are demonstrated in Table 7. Based on chemometric results, a significant impact of *C. butyricum* probiotic feed additive on the variation in milk FA profiles was continually remarkable throughout the entire lactation period of the sows. 

### 3.5. Changes in Non-Volatile Polar Metabolite Profiles of Sow Colostrum and Milk

**Figure 3:**
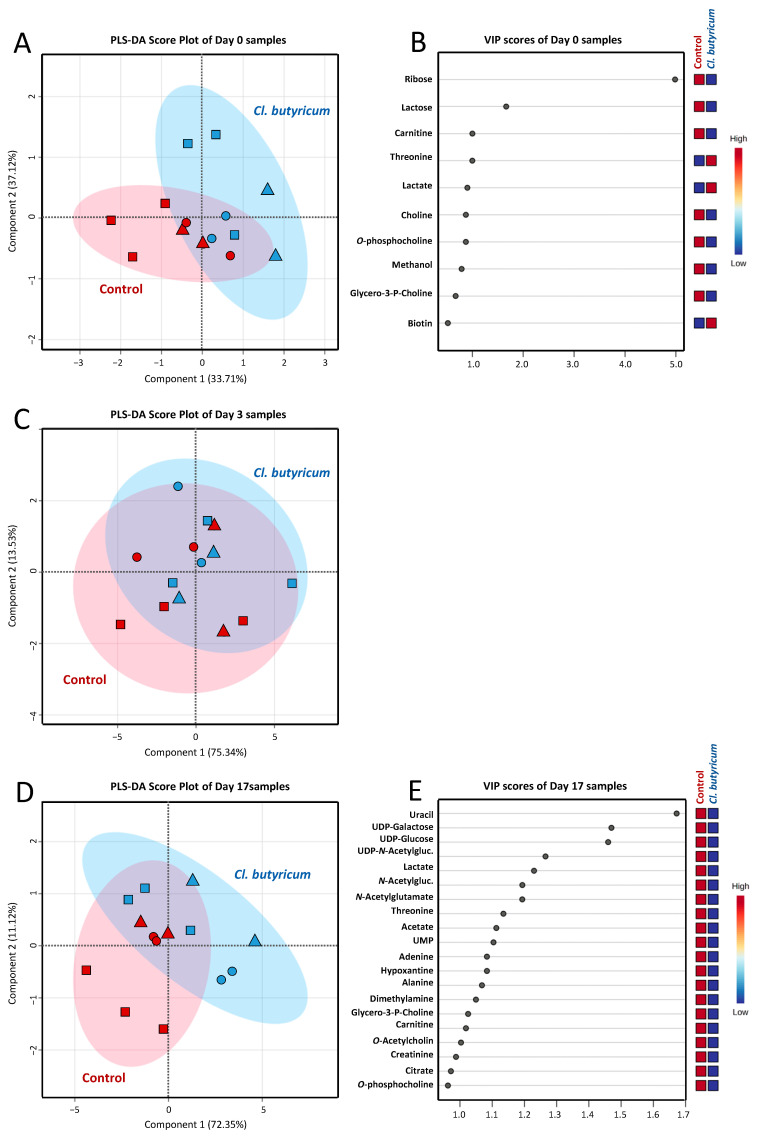
PLS-DA score plots for the comparison of non-volatile polar metabolite profiles of colostrum (day 0; panel (**A**)), transient milk (day 3; panel (**C**)) and mature milk (day 17; panel (**D**)) samples collected from sows in the control (red color) and *C. butyricum* treatment (blue color) group. Samples from animals in parity 1 (■), parity 2–4 (▲) and parity 5–7 (●) are differently symbolized. VIP scores derived from the comparison among samples of the same day postpartum and indicative metabolites accountable for the discrimination are visualized in panel (**B**) and (**E**), respectively.

Non-volatile polar metabolites including amino acids, carbohydrates, alcohols, organic acids and lipid derivatives in colostrum and milk samples were identified by a non-targeted ^1^H-NMR analysis. As mentioned in FA profile analysis, three separate PLS-DAs were performed for comparison of non-volatile polar metabolite profiles of colostrum (day 0), transient (day 3) and mature milk (day 17) samples (Figure 3). Regarding colostrum, PLS-DA demonstrated a good distinction pattern between the control and *C. butyricum* treatment group, with a prediction accuracy of 78.57%, *R*^2^ = 0.604 and *Q*^2^ = 0.561 (Figure 3A). VIP scores indicated that variations in the concentration of ribose, lactose, carnitine, threonine, lactate, choline and o-phosphocholine were responsible for the discrimination (Figure 3B). In the case of transient milk, it was remarkable that the distinction between the two groups of samples disappeared during this transition period (Figure 3C). Interestingly, however, a clear distinction between the control and *C. butyricum* treatment group returned to be remarkable in mature milk again. This change in metabolite pattern was demonstrated by PLS-DA with a prediction accuracy of 71.42%, *R*^2^ = 0.721 and *Q*^2^ = 0.540 (Figure 3D). VIP scores indicated that variations in the concentration of uracil, UDP-galactose, UDP-glucose, UDP-*N*-Acetylglucosamine, lactate, *N*-Acetylglucosamine, *N*-acetylglutamate, threonine, acetate, UMP, adenine, hypoxantine, alanine, dimethylamine, glycero-3-P-choline, carnitine, O-acetylcholine and creatinine were responsible for the discrimination in mature milk from the control and *C. butyricum* treatment groups (Figure 3E). Variations in the concentrations of indicative non-volatile polar metabolites in sow colostrum, transient and mature milk—with their statistically significant levels—are demonstrated in Table 8. Based on the overall metabolite pattern recognition, a significant impact of dietary *C. butyricum* probiotic additive was only observed in colostrum and mature milk.

## 4. Discussion

The *C. butyricum* has benefits for animal health; i.e., improving gut microbiota and intestinal barrier functions leading to increase the growth performances of both sows and piglets [4,5]. Relative backfat loss serves as an important parameter that indicates sow energy mobilization. It should be noted that backfat thickness is associated with a variety of reproductive parameters. For example, loss of backfat thickness could increase the number of stillborn piglets and decrease litter size [27]. Moreover, excessive backfat loss during lactation is associated with prolonged weaning-to-estrus intervals and reduced farrowing rates [28,29]. It is recognized that 15–20% of primiparous sows are often culled due to reproductive problems [30,31,32]. The present study found that the application of dietary *C. butyricum* probiotic additive during late gestation throughout the entire lactating period of the sows could reduce relative backfat loss, especially in primiparous sows. It has been documented that probiotics enhance intestinal barrier function and enzymatic production, which leads to improved nutrient digestion, health, and reproductive performances of the sows [4,33,34]. *C. butyricum* probiotics are noted to have a capacity to produce short-chain fatty acids (SCFAs) such as butyrate from carbohydrate metabolism, that could provide energy for epithelial cells and promote intestinal barrier function [35,36]. Furthermore, Niu et al. [37] demonstrated the association between a high abundance of *Clostridium* spp. in the gastrointestinal tract and higher backfat thickness. Therefore, feeding sows with *C. butyricum* probiotic additives may affect energy metabolization and digestion. Indeed, our result was consistent with the work of Konieczka et al. [38], who also found that feed formulation with probiotic *Bacillus subtilis* and *Bacillus amyloliquefaciens* could reduce backfat thickness loss in sows during lactation. 

In the present study, the average body weight of all piglets at birth and during lactation was not affected by the *C. butyricum* probiotic additive to the sow’s diet during late gestation to lactation. Nevertheless, the piglets of sows fed with *C. butyricum* incorporated in their diets had higher body weights on days 21 than those of the control group. *C. butyricum* probiotics have been found to enhance the growth performance of suckling piglets by improving milk quality and increasing the lactose and protein content in milk [39]. In contrast, in our study, the *C. butyricum* probiotic additive only tended to improve the lactose content in mature milk (day 17; *p* = 0.074), which is a source of energy for piglet metabolism. 

Direct feeding of *C. butyricum* probiotics has been shown to promote the growth performance of piglets by improving enterocyte morphology, increasing villus height, improving the villus height–cell depth ratio and strength of the intestinal mucosa cell wall, enabling better nutrient absorption and a reduced diarrhea score [40,41]. Moreover, López et al. (2021) found that *C. butyricum* increased intestinal butyric acid levels, thereby improving the intestinal wellness and health status [42]. In agreement with the present study, enhanced growth coinciding with the reduction of diarrhea scores in pre-weaning piglets was also observed. However, it could be an indirect effect of lactating sows fed with probiotic *C. butyricum* incorporated diets. The results showed that piglets born from *C. butyricum*-fed sows had high body weights at day 21 when compared to the control group. Moreover, feeding *C. butyricum* probiotics to sows reduced the incidence of diarrhea in piglets throughout the lactation period. Pre-weaning piglet mortality is primarily caused by crushing, low viability and diarrhea [43,44]. Therefore, preventing diarrhea in suckling piglets is essential to minimize these problems. Probiotic additives have been shown to reduce piglet diarrhea both before weaning [45,46] and after weaning [41,42]. In general, the *C. butyricum*—a butyric acid-producing bacterium—lowers gut pH, which enhances antibacterial effects [47,48]. Cao et al. [39] indicated that *C. butyricum* probiotic additive promotes an intestinal microecology that supports beneficial bacteria subpopulations, such as *Bacteroidetes* and *Prevotellaceae* spp., while reducing harmful bacteria, including *Streptococcus*, *Escherichia*, and *Shigella*, in pre-weaning piglets. Tang et al. [5] further indicated that supplementing with this probiotic strain significantly reduces the colonization of harmful bacteria in the intestinal tract and enhances the expression of tight junctions (TJs) to improve intestinal barrier function. Furthermore, a reduction in serum lipopolysaccharides endotoxin concentrations, the major factors that induce inflammation and disrupt TJ protein, was found in sows fed with *C. butyricum* probiotics on day 21 of lactation [39]. This is in agreement with Kong et al. [49] who reported that consumption of *C. butyricum* probiotics benefited the gastrointestinal tract microbiome by increasing the beneficial bacteria and reducing harmful pathogens in the intestines of children [50]. Although *C. butyricum* probiotic additive in the present study was applied in the diets of sows, significant improvement in diarrhea incidence was remarkable in piglets. This might be due to the bacteria from the sows which colonized the mammary teats and were transferred to the piglets during suckling a few days after birth [34,41]. Therefore, the addition of *C. butyricum* probiotics additive to the diet of sows may increase the concentration of butyric acid in the gastrointestinal tract of the piglets. As a result, piglets from sows receiving probiotics have a lower incidence of diarrhea and gain a higher body weight. This improvement in digestive function and absorptive capacity of the intestine contributes to the overall health and growth of the piglets. 

The significant effect of dietary *C. butyricum* probiotic additive was neither observed on gross chemical composition nor immunological quality of colostrum and milk of the sows in the probiotic-treated group in this study. This was in accordance with two studies which previously reported no significant influence of probiotic administration—i.e., *C. butyricum* [34] and *Bacillus licheniformis* and *Bacillus subtilis* [51]—on the contents of fat, protein, and lactose in sow colostrum. However, both studies found significant changes in fat, protein and lactose content in the mature milk of sows fed with probiotics. Indeed, many studies have reported significant impacts of probiotic administration—i.e., *Bacillus subtilis* and *Bacillus licheniformis* [8,13], *Saccharomyces boulardii* [7], *Saccharomyces cerevisiae* [14]—on the variations in fat, protein, lactose and dry matter content in both colostrum and mature milk of sows. Regarding immunological parameters, significant rises in IgG, IgA and IgM level in sow colostrum were found after probiotic administration in many studies [8,14,45]. In the present study, the effect of dietary *C. butyricum* probiotic additive on the colostral immunoglobulin contents could not be observed. It should be mentioned that inconsistent and very divergent results have been documented regarding the scientific benefits of probiotics when applied in the field. This can be related to the variations of probiotic strains, dosage and delivery methods; sow individuality and herd; animal housing and farming practices, as well as other environmental factors linked to specific swine production systems [4]. In addition to there being no significant effect on gross chemical and immune components, fatty acid and non-volatile polar metabolite profiling was performed to provide more insights on the impact of dietary *C. butyricum* probiotic additive on the metabolome of sow colostrum, transient and mature milk in this study. 

It is well recognized that nutritional strategies during late gestation and the lactation period can induce changes in FA composition in sow colostrum and milk [52]. Alterations in milk FA profiles linked to probiotic administration have been reported in goats [53], ewes [54] and dairy cows [55]. However, information regarding the effect of probiotic administration on FA modification in sow milk is rather limited. Our results demonstrated a substantial impact of dietary *C. butyricum* probiotic additive on sow milk FA profiles. A continued distinction pattern of FA profiles between the control and *C. butyricum* treatment groups was observed in the colostrum, transient and mature milk of the sows. PLS-DA derived VIP scores suggested that variations in the concentration of: (i) saturated fatty acids (SFA) including caprylic (C8:0), capric (C10:0), lauric (C12:0), arachidic (C20:0) and behenic (C22:0) acid; (ii) monounsaturated fatty acids (MUFA) including myristoleic (C14:1), palmitoleic (C16:1) and paullinic (C20:1n7) acid; and (iii) polyunsaturated fatty acids (PUFA) including linolenic (C18:3n3), eicosadienoic (C20:2n6), eicosatrienoic (C20:3n3), docosatetraenoic (C22:4), DPA (22:5n3) and docosahexaenoic (C22:6n3) acid were accountable for the discrimination. Although chemometric analysis revealed a good distinction pattern in colostrum and milk FA profiles associated with probiotic *C. butyricum* consumption, the *p* values of individual FAs were not statistically significant (*p* > 0.05). The most prominent increase in palmitoleic acid (C16:1) was detected in colostrum (*p* = 0.059) and transient milk (*p* = 0.006) of the sows in the treatment group. Also, the concentrations of lauric (C12:0), myristoleic (C14:1) and linolenic (C18:3n3) acid tended to increase in the transient and mature milk of the sows in the *C. butyricum* treatment group. The higher levels in the milk FA could be attributed to improved nutrient digestion and absorption of the sows induced by the *C. butyricum* probiotics. Increasing trends of FAs in sow milk were also observed after probiotic yeast intake in the study of Domingos et al. [56]. It has been documented that certain medium- and long-chain FAs have promising antibacterial activities along with bioactivities to enhance epithelial barrier functions and gut health [57,58]. Therefore, a higher abundance of these FAs in milk might be linked to the reduction of diarrhea scores in pre-weaned piglets belonging to the sows in the probiotics treatment group of this study. 

The application of non-targeted ^1^H-NMR metabolomics is well acknowledged in lactation research. The advantage is to provide comprehensive characterization of overall metabolites present in milk and their modifications under different conditions [59]. Information regarding changes in milk metabolite profiles after probiotic administration has been reported in livestock such as dairy cows [60], donkeys [61] and sows [6,7,8,19,45,56]. Additionally, parity numbers and lactation stages were found to be the main factors influencing the composition of metabolomics in colostrum and milk in both monogastric and non-monogastric animals [18,62]. Our results demonstrated a substantial impact of dietary *C. butyricum* probiotic additive on the metabolite profiles of sow milk. A good distinction between the control and *C. butyricum* treatment groups was notably observed in colostrum and mature milk. PLS-DA-derived VIP scores suggested that variations in the concentration of certain carbohydrates, amino acids, amines, organic acids as well as their derivatives were accountable for the discrimination. It should be noted that the concentration of most indicative metabolites significantly decreased (*p* < 0.05)—i.e., ribose, dimethylamine, *N*-acetylglutamate, O-acetylcholine, sn-glycero-3-phosphocoline, UDP-galactose, UDP-glucose, UDP-*N*-acetylglucosamine and uracil—or tended to change (*p* < 0.10)—i.e., lactose, lactate, carnitine, choline, *N*-acetylglucosamine, *O*-phosphocholine and threonine—in the milk of sows in the probiotics treatment group. The influence of the dietary probiotic *Bacillus subtilis* and *Bacillus amyloliquefaciens* applications on sow milk metabolome has been reported by Saladrigas-García et al. [19]. In their study, variations in milk metabolites—especially lactose, UDP-*N*-acetylglucosamine, creatine phosphate, UDP-galactose and glycoprotein—were found to be associated with administration of the tested probiotic *Bacillus* strains. In this study, similar indicative metabolites—i.e., lactose, UDP-N-acetylglucosamine, and UDP-galactose—were observed in association with the use of the probiotic, *C. butyricum*. Another recent study focusing on the impact of multispecies probiotics (SLAB51)—consisting of *Streptococcus thermophilus*, *Bifidobacterium lactis*, *Lactobacillus brevis*, *Lactobacillus plantarum*, *Lactobacillus paracasei*, *Lactobacillus acidophilus*, and *Lactobacillus helveticus*—administration on donkey milk metabolome found significant changes in the concentration of 12 metabolites—i.e., lactose, *O*-phosphocholine, sn-glycero-3-phosphocholine, 4-pyridoxate, caprylate, isovalerate, butyrate, 2-oxoisocaproate, glucose, glucose-1-phosphate, glutamine, and 4-hydroxyphenilacetate—in donkey milk (*p* < 0.05) induced by administration of the probiotic mixture SLAB51 [60]. Moreover, this study found a decreasing trend in the concentration of lactate (*p* = 0.16) and threonine (*p* = 0.14) in donkey milk induced by SLAB51 probiotics. Alterations in lactose, lactate, threonine, *O*-phosphocholine and sn-Glycero-3-phosphocholine contents were also linked to the application of the *C. butyricum* probiotics in our work. Nevertheless, it should be mentioned that the beneficial effects of dietary probiotic administration on milk production and compositional changes reported in various livestock species are very case-specific and still inconclusive. Inconsistent findings and great variability in results could be due to probiotic-specific factors—e.g., probiotic strains and mixed formulation, dosage, mode and duration of administration to the subject animals—as well as host-specific factors; e.g., animal species and breed, health and physiological status, digestive system and gut health, diet composition and feeding regime [60]. Modifications in milk composition could be mediated by changes in digestive efficiency, nutrient absorption and metabolic response of the sows induced by probiotics and other beneficial microbes in their gut microbiota [19]. Therefore, further research is needed to better understand the mechanisms by which probiotic administration can impact milk composition, and to determine the optimal dosages and feeding durations for various probiotic strains. Moreover, the impact of probiotics on the fecal microbiota of sows and piglets is another point of interest that requires further investigation. 

## 5. Conclusions

The present study demonstrated the impacts of *C. butyricum* probiotic feed additive on sow and piglet performances, together with the alterations in lipidomic and metabolomic profiles of sow milk. The results revealed that diets with *C. butyricum* probiotic additive provided no significant impact on the overall reproductive performance of sows. However, it was remarkable that *C. butyricum* probiotic additive resulted in a significantly lower backfat loss in primiparous sows and a significant increase in piglet weight at day 21 of lactation in parity 5–7 sows. In addition, the piglets from sows fed with probiotic *C. butyricum*-added diets experienced significantly lower diarrhea scores throughout the lactation period. In addition to animal performances, *C. butyricum* probiotic additive also induced notable changes in lipidomic and metabolomics profiles of sow colostrum and mature milk. Significant variations in the concentration of certain indicative fatty acids and metabolite compounds indicated a notable impact on the nutritional profile of sow milk. In conclusion, the use of *C. butyricum* probiotics in sows may improve sow body condition and reduce diarrhea incidence in piglets, with underlying changes in milk composition that warrant further investigation. These findings support the potential of *C. butyricum* probiotics as a beneficial feed additive in swine production.

## Figures and Tables

**Table 1:** Nutritional content of the experimental diets during the gestation and lactation period.

Nutritional Content	Gestation Diet	Lactation Diet
Metabolizable energy, kcal/kg	2783	3363
Crude protein, %	11.76	16.21
Crude fat, %	5.75	8.39
Crude fiber, %	4.63	4.70
Ash, %	11.26	7.53
Moisture, %	9.72	9.97
Lysine, %	1.50	2.60

**Table 2:** Effect of dietary *C. butyricum* probiotic additive on sow reproductive performances (*n* = 64 sows). Values are the least-squares means of samples ± SEM.

Parameters	Control	Treatment	*p* Value
Number of sows, sows	35	29	
Sow parity number	3.2 ± 0.1	3.1 ± 0.1	0.726
Number of totals born piglet/litter, piglets	12.3 ± 0.5	12.9 ± 0.6	0.501
Number of live born piglet/litter, piglets	10.8 ± 0.5	11.3 ± 0.6	0.488
Percentage of stillborn piglet/litter, %	7.1 ± 2.6	8.4 ± 2.9	0.724
Percentage of mummified fetus/litter, %	4.0 ± 1.8	2.8 ± 2.0	0.668
Number of weaned piglet/litter, piglets	7.1 ± 0.6	6.9 ± 0.6	0.757
Litter weight at weaning, kg	47.7 ± 3.8	46.5 ± 3.6	0.802
Backfat thickness at farrowing, mm	21.4 ± 1.0	20.9 ± 1.1	0.732
Backfat thickness at weaning, mm	18.6 ± 0.7	18.1 ± 0.8	0.612
Backfat loss during lactation (%)	9.6 ± 2.4	5.0 ± 2.8	0.215
Milk yield between Day 3 and 10 of lactation, kg	7.6 ± 0.4	7.5 ± 0.4	0.822
Milk yield between Day 10 and 17 of lactation, kg	7.8 ± 0.5	7.5 ± 0.4	0.651
Litter weight gain between Day 3 and 10 of lactation, kg	1.5 ± 0.2	1.1 ± 0.2	0.078
Litter weight gain between Day 10 and 17 of lactation, kg	0.8 ± 0.2	0.8 ± 0.2	0.817
Piglet preweaning mortality between Day 3–21, %	28.9 ± 4.7	27.6 ± 4.6	0.846

**Table 3:** Effect of dietary *C. butyricum* probiotic additive on sow reproductive performances by parity. Values are the least-squares means of samples.

Parameters	Parity					
	1		2–4		5–7	
	Control	Treatment	Control	Treatment	Control	Treatment
Number of total born piglet/litter, piglets	12.1	13.7	12.3	11.9	12.6	13.0
Number of live born piglet/litter, piglets	9.4	11.4	11.1	10.4	11.9	12.2
Percentage of stillborn piglet/litter, %	12.1	7.1	7.0	12.7	2.1	5.5
Percentage of mummified fetus/litter, %	7.0	7.9	1.7	0.6	3.2	0.1
Number of weaned piglet/litter, piglets	6.3	5.2	6.9	8.2	8.2	7.2
Litter weight at weaning, kg	41.7	32.9	45.3	55.3	56.2	51.2
Backfat loss during lactation (%)	11.6 ^b^	−2.3 ^a^	12.0	5.0	5.2	12.3
Milk yield between Day 3 and 10 of lactation, kg	6.4	7.8	8.1	7.6	8.2	7.0
Milk yield between Day 10 and 17 of lactation, kg	7.0	7.5	7.4	7.8	8.9	7.1
Litter weight gain between Day 3 and 10 of lactation, kg/day	1.1	0.6	1.7	1.6	1.8	1.0
Litter weight gain between Day 10 and 17 of lactation, kg/day	0.8	0.8	0.9	0.8	0.9	0.7
Piglet preweaning mortality between Day 3–21, %	30.6	36.7	28.1	20.1	28.1	26.0

^a,b^ Different superscript letters within the same parity class indicate a significant difference at *p* < 0.05.

**Table 4:** Pre-weaning piglet performances in control (n = 426) and treatment (n = 368) groups and at different parity classes of the sows. Values are the least-squares means of samples.

Parameters	Group				Parity				
	Control	Treatment	SEM	*p* Value	1	2–4	5–7	SEM	*p* Value
Piglet birth weight, kg	1.45	1.43	0.07	0.818	1.18 ^b^	1.59 ^a^	1.56 ^a^	0.08	<0.001
Piglet weight at 24 h after birth, kg	1.56	1.50	0.06	0.506	1.30 ^b^	1.68 ^a^	1.61 ^a^	0.08	<0.001
Piglet weight at Day 3, kg	1.79	1.80	0.06	0.914	1.64 ^b^	1.92 ^a^	1.82 ^ab^	0.09	0.006
Piglet weight at Day 21, kg	3.87	4.32	0.19	0.085	3.84 ^b^	4.54 ^a^	3.92 ^ab^	0.29	0.023

^a,b^ Different superscript letters within the same row indicate a significant difference at *p* < 0.05. SEM = standard error of mean.

**Table 5:** Pre-weaning piglet performances in control (*n* = 426) and treatment (*n* = 368) groups by different parity classes of the sows. Values are the least-squares means of samples.

Parameters	Parity								
	1			2–4			5–7		
	Control	Treatment	SEM	Control	Treatment	SEM	Control	Treatment	SEM
Piglet birth weight, kg	1.29	1.08	0.13	1.62	1.55	0.11	1.45	1.67	0.11
Piglet weight at 24 h after birth, kg	1.37	1.22	0.11	1.72	1.64	0.11	1.59	1.64	0.11
Piglet weight at Day 3, kg	1.70	1.57	0.10	1.90	1.94	0.08	1.76	1.88	0.14
Piglet weight at Day 21, kg	3.84	3.84	0.31	4.49	4.58	0.25	3.29 ^b^	4.55 ^a^	0.40

^a,b^ Different superscript letters within the same row and the same class of parity indicate a significant difference at *p* < 0.05. SEM = standard error of mean.

**Table 6:** Effect of dietary *C. butyricum* probiotic additive on the variations in major chemical composition of sow colostrum (day 0), transient milk (day 3) and mature milk (day 17). Values are the least-squares means of samples.

Composition	Day 0				Day 3				Day 17			
	Con	Treat	SEM	*p* Value	Con	Treat	SEM	*p* Value	Con	Treat	SEM	*p* Value
Fat, g/100 g	5.99	5.53	0.49	0.492	11.13	9.97	0.64	0.222	8.13	7.62	0.26	0.159
Protein, g/100 g	15.80	17.10	0.12	0.118	5.73	5.90	0.31	0.722	5.20	4.96	0.16	0.273
Lactose, g/100 g	2.437	2.366	0.67	0.666	4.25	4.39	0.08	0.260	4.46	4.71	0.10	0.074
DM, g/100 g	24.98	25.66	0.51	0.506	22.80	21.86	0.75	0.398	19.56	19.13	0.32	0.335
Casein, g/100 g	12.58	13.70	0.10	0.103	4.169	4.26	0.16	0.695	4.04	4.07	0.08	0.813
IgG, mg/mL	42.02	40.80	0.76	0.763	NA	NA	NA	NA	NA	NA	NA	NA
IgA, mg/mL	10.23	10.71	0.78	0.779	NA	NA	NA	NA	NA	NA	NA	NA

Con = control group, Treat = treatment group, SEM = standard error of mean. NA = not applicable.

**Table 7:** Effect of dietary *C. butyricum* probiotic additive on the variations of indicative fatty acids in sow colostrum (day 0), transient milk (day 3) and mature milk (day 17). Values are the least-squares means of samples.

Fatty Acid	Day 0				Day 3				Day 17			
	Con	Treat	SEM	*p* Value	Con	Treat	SEM	*p* Value	Con	Treat	SEM	*p* Value
Caprylic acid	0.011 *	0.014	0.005	0.708	0.013	0.013	0.004	0.967	0.029	0.033	0.004	0.512
Capric acid	0.122	0.017	0.062	0.265	0.060	0.070	0.027	0.816	0.182	0.199	0.028	0.673
Lauric acid	1.521	1.018	0.321	0.300	1.642	1.770	0.467	0.851	3.067	3.185	0.504	0.874
Myristoleic acid	-	0.033	0.007	0.672	0.104	0.113	0.035	0.973	0.284	0.333	0.052	0.520
Palmitoleic acid	1.649	1.958	0.100	0.059	2.625 ^a^	1.254 ^b^	0.260	0.006	1.642	0.580	0.091	0.641
Cis-10-heptadecarnoic acid	0.114	0.132	0.009	0.156	0.177	0.192	0.018	0.570	0.156	0.187	0.014	0.148
Linoleic acid	26.42	24.46	0.863	0.147	19.07	19.88	0.574	0.345	19.410	18.652	0.869	0.555
Arachidic acid	0.167	0.165	0.018	0.928	0.196	0.182	0.010	0.346	0.190	0.166	0.011	0.164
Paullinic acid	0.300	0.366	0.030	0.121	0.627	0.548	0.065	0.414	0.655	0.408	0.109	0.147
Linolenic acid	1.804	1.735	0.160	0.769	1.377	1.386	0.097	0.944	1.300	1.475	0.147	0.426
Eicosadienoic acid	0.519	0.592	0.045	0.284	0.646	0.585	0.051	0.424	0.463	0.400	0.053	0.427
Eicosatrienoic acid	0.322	0.124	0.122	0.285	0.114	0.113	0.010	0.946	0.084	0.104	0.012	0.264
Docosatetraenoic acid	0.244	0.285	0.022	0.230	0.217	0.202	0.025	0.688	0.220	0.186	0.030	0.457
Docosapentaenoic acid	0.357	0.367	0.03	0.788	0.378	0.272	0.073	0.334	0.450	0.246	0.098	0.181

Con = control group, Treat = treatment group, SEM = standard error of mean. * Fatty acid contents are expressed as mg/100 g. ^a,b^ Different superscript letters within the same row indicate a significant difference at *p* < 0.05.

**Table 8:** Effect of dietary *C. butyricum* probiotic additive on the variations of indicative non-volatile polar metabolites in sow colostrum (day 0), transient milk (day 3) and mature milk (day 17). Values are the least-squares means of samples.

Metabolite	Day 0				Day 3				Day 17			
	Con	Treat	SEM	*p* Value	Con	Treat	SEM	*p* Value	Con	Treat	SEM	*p* Value
Carnitine	9.260 *	9.175	0.038	0.154	8.527	8.523	0.062	0.967	8.016	7.862	0.047	0.052
Choline	9.301	9.225	0.037	0.178	8.571	8.562	0.065	0.925	8.054	7.908	0.046	0.055
Dimethylamine	8.611	8.624	0.033	0.774	7.809	7.773	0.068	0.713	7.336 ^a^	7.175 ^b^	0.048	0.045
Lactate	8.809	8.864	0.030	0.227	7.897	7.832	0.095	0.643	7.442	7.258	0.059	0.057
Lactose	9.837	9.714	0.043	0.081	10.169	10.209	0.052	0.601	10.602	10.899	0.040	0.567
*N*-Acetylglucosamine	9.418	9.425	0.033	0.896	8.626	8.575	0.077	0.650	8.122	7.945	0.055	0.053
*N*-Acetylglutamate	9.283	9.287	0.037	0.941	8.531	8.490	0.078	0.720	8.065 ^a^	7.889 ^b^	0.052	0.044
*O*-Acetylcholine	9.436	9.389	0.037	0.384	8.676	8.665	0.065	0.912	8.185 ^a^	8.034 ^b^	0.034	0.041
*O*-Phosphocholine	9.301	9.225	0.037	0.178	8.571	8.562	0.065	0.925	8.054	7.907	0.046	0.055
Ribose	6.339 ^a^	5.982 ^b^	0.089	0.022	6.536	6.397	0.237	0.688	7.448	7.410	0.056	0.644
sn-Glycero-3-phosphocoline	9.466	9.405	0.038	0.284	8.715	8.702	0.066	0.888	8.208 ^a^	8.056 ^b^	0.046	0.045
Threonine	8.617	8.680	0.029	0.169	7.687	7.624	0.094	0.654	7.237	7.066	0.060	0.078
UDP-Galactose	8.359	8.336	0.037	0.669	7.627	7.550	0.084	0.533	7.186 ^a^	6.966 ^b^	0.057	0.026
UDP-Glucose	8.454	8.435	0.037	0.725	7.693	7.630	0.083	0.607	7.265 ^a^	7.047 ^b^	0.055	0.023
UDP-*N*-Acetylglucosamine	9.059	9.063	0.034	0.937	8.359	8.322	0.073	0.731	7.915 ^a^	7.723 ^b^	0.050	0.026
Uracil	8.170	8.139	0.042	0.616	7.394	7.328	0.108	0.677	7.057 ^a^	6.817 ^b^	0.054	0.014

Con = control group, Treat = treatment group, SEM = standard error of mean. * Non-volatile polar metabolite contents are expressed as log_10_ transformed [arbitrary unit]. ^a,b^ Different superscript letters within the same row indicate a significant difference at *p* < 0.05.

## Data Availability

Data produced in this study are available from the corresponding authors on reasonable request.
